# Transplant Trial Watch

**DOI:** 10.3389/ti.2021.10216

**Published:** 2022-01-26

**Authors:** John M. O’Callaghan

**Affiliations:** ^1^ University Hospitals Coventry and Warwickshire, Coventry, United Kingdom; ^2^ Centre for Evidence in Transplantation, University of Oxford, Oxford, United Kingdom

**Keywords:** kidney transplantation, randomised controlled trial, cytomegalovirus, mesenchymal stromal cells, prophylaxis

**Randomised Controlled Trial 1**

**Immunoguided Discontinuation of Prophylaxis for Cytomegalovirus Disease in Kidney Transplant Recipients Treated with Antithymocyte Globulin: A Randomized Clinical Trial**

*by Paez-Vega, A., et al. Clinical Infectious Diseases 2021 [record in progress].*

**Randomised Controlled Trial 2**

**Autologous Bone Marrow-Derived Mesenchymal Stromal Cell Therapy With Early Tacrolimus Withdrawal: The Randomized Prospective, Single-Center, Open-Label TRITON Study**

*by Reinders, M. E. J., et al. American Journal of Transplantation 2021; 21 (9): 3055–3065.*

To keep the transplantation community informed about recently published level 1 evidence in organ transplantation ESOT and the Centre for Evidence in Transplantation have developed the Transplant Trial Watch. The Transplant Trial Watch is a monthly overview of 10 new randomised controlled trials (RCTs) and systematic reviews. This page of Transplant International offers commentaries on methodological issues and clinical implications on two articles of particular interest from the CET Transplant Trial Watch monthly selection. For all high quality evidence in solid organ transplantation, visit the Transplant Library: www.transplantlibrary.com

RANDOMISED CONTROLLED TRIAL 1 Immunoguided Discontinuation of Prophylaxis for Cytomegalovirus Disease in Kidney Transplant Recipients Treated with Antithymocyte Globulin: A Randomized Clinical Trial

*by Paez-Vega, A., et al. Clinical Infectious Diseases 2021 [record in progress].*

## Aims

This study aimed to assess if it is safe and effective to terminate antiviral prophylaxis when cytomegalovirus (CMV)- specific cell-mediated immunity (CMI) is detected following induction treatment and to continue with preemptive therapy (immunoguided prevention), in renal transplant patients.

## Interventions

Participants were randomly assigned to either immunoguided prevention or fixed-duration prophylaxis.

## Participants

One-fifty CMV-seropositive kidney transplant patients.

## Outcomes

Incidence of CMV disease and replication.

## Follow-up

Tweleve months

## CET Conclusion

This non-inferiority design trial randomized kidney transplant recipients to either fixed-duration CMV prophylaxis, or CMV cell-immunity guided prophylaxis. The authors report both strategies to be equivalent, supporting the idea that prophylaxis can be terminated early in patients with restored cellular immunity to CMV. Immunoguided prophylaxis resulted in less neutropenia. The study design is robust and provides good evidence that CMI-guided prophylaxis is safe and effective in this low-risk population of CMI positive and seropositive patients. Future studies will be needed to evaluate generalizability to other populations, and to establish cost-effectiveness.

## Jadad Score

3.

## Data Analysis

Per protocol analysis.

## Allocation Concealment

Yes.

## Trial Registration

ClinicalTrials.gov—NCT03123627

## Funding Source

Non-industry funded.

RANDOMISED CONTROLLED TRIAL 2 Autologous Bone Marrow-Derived Mesenchymal Stromal Cell Therapy With Early Tacrolimus Withdrawal: The Randomized Prospective, Single-Center, Open-Label TRITON Study

*by Reinders, M. E. J., et al. American Journal of Transplantation 2021; 21 (9): 3055–3065.*

## Aims

The aim of this post hoc analysis was to investigate the effect of mesenchymal stromal cell (MSC) therapy with early tacrolimus withdrawal in renal transplant patients.

## Interventions

Participants in the original trial were randomised to either MSC plus early tacrolimus withdrawal or to standard tacrolimus dose.

## Participants

Seventy living donor kidney transplant recipients.

## Outcomes

The primary outcome was quantitative assessment of interstitial fibrosis. The secondary outcomes were patient death, graft loss, acute rejection, renal function, adverse events, and immunological responses.

## Follow-up

Five years.

## CET Conclusion

This is a good quality and fair sized randomised controlled trial in renal transplantation. Patients received alemtuzumab induction therapy and then were maintained on prednisolone and tacrolimus. In the study arm, patients also received autologous mesenchymal stem cell (MSC) infusions and then had tacrolimus minimisation and subsequent withdrawal. The intention was to see if fibrosis could be reduced through tacrolimus withdrawal, using MSCs to reduce the risk of rejection in this context. Randomisation was performed by an online system and is likely to be truly random, however the nature of the intervention means that the study was not easily blinded and there is the potential for bias. However, pathologists examining the biopsies were blinded to the allocation and used standardised scoring, which is an important strength of the study. Withdrawals and dropouts are adequately described and the statistical methods are appropriate. The analysis was however not by strict intention-to-treat; one in 12 patients allocated to the study arm had abnormal MSC growth and could not receive that intervention so were excluded from the analysis for example. There were four patients in the control arm who refused to have a follow up biopsy and so were also excluded. These seem small numbers, but in a small trial are significant. The overall fibrosis scores and progression of fibrosis was the same in both arms of the study. Renal function was similar and risk of acute rejection was similarly low between the study arms. There was a significantly higher number of Tregs in the MSC group. A post hoc analysis of 5-years outcomes is presented, but does not indicate any significant differences. The study was too small to identify any significant difference in graft or patient survival. In conclusion, the use of MSC was safe within this study and was not associated with increased risk of rejection when combined with tacrolimus withdrawal.

## Jadad Score

3.

## Data Analysis

Per protocol analysis.

## Allocation Concealment

Yes.

## Trial Registration

ClinicalTrials.gov—NCT02057965.

## Funding Source

Industry funded.

## Clinical Impact Summary

This study from the Netherlands is a good quality randomised controlled trial in renal transplantation and it supports the ongoing investigation of mesenchymal stem cells (MSC) as a potential component of immune suppression regimens.

Renal transplant recipients in the trial received alemtuzumab induction therapy and then were maintained on prednisolone and tacrolimus. In the study arm patients also received two infusions of autologous mesenchymal stem cells (MSCs) and then progressed to tacrolimus minimisation and subsequent withdrawal. The intention was to see if fibrosis could be reduced through tacrolimus withdrawal, using MSCs to safely reduce the risk of rejection in this context. The study was necessarily open-label to the patient and clinicians. However, pathologists examining the biopsies were blinded to the allocation and used standardised scoring, this is an important strength of the study.

Blinded assessment of biopsy scores was similar for both groups and showed similar progression over 24 weeks. There was only one episode of acute rejection in the MSC group on for-cause biopsy and none in the control arm. This was present in a patient on reduced immune suppression due to BK virus infection. There was no graft or patient loss in either arm, but the study was too small to really assess for these outcomes. Protocol biopsies showed a mixture of TCMR and ABMR in three to four patients in each study group during the study period. There were no serious adverse events directly related to the infusion of MSCs and the overall adverse event rate was similar between the study arms.

Whilst there was no statistically significant difference between the groups in terms of most leukocyte cell lines quantified, there was a significant increase in Tregs in the MSC group that persisted up to 52 weeks after transplantation.

The study was too small to identify any significant difference in graft or patient survival, particularly at later timepoints. In conclusion, the use of MSC was safe within this study and was not associated with increased risk of rejection when combined with tacrolimus withdrawal. Whilst there was no apparent difference in fibrosis on biopsy scores, the increase in Tregs is intriguing and there is a potential to see improved GFR at longer follow up in a larger study. This is an exciting potential avenue to improve long-term allograft survival and warrants further exploration in a larger study.

